# Recognizing speculative language in biomedical research articles: a linguistically motivated perspective

**DOI:** 10.1186/1471-2105-9-S11-S10

**Published:** 2008-11-19

**Authors:** Halil Kilicoglu, Sabine Bergler

**Affiliations:** 1 Department of Computer Science and Software Engineering, 1455 de Maisonneuve Blvd West, H3G 1M8, Montréal, Québec, Canada

## Abstract

**Background:**

Due to the nature of scientific methodology, research articles are rich in speculative and tentative statements, also known as hedges. We explore a linguistically motivated approach to the problem of recognizing such language in biomedical research articles. Our approach draws on prior linguistic work as well as existing lexical resources to create a dictionary of hedging cues and extends it by introducing syntactic patterns.

Furthermore, recognizing that hedging cues differ in speculative strength, we assign them weights in two ways: automatically using the information gain (IG) measure and semi-automatically based on their types and centrality to hedging. Weights of hedging cues are used to determine the speculative strength of sentences.

**Results:**

We test our system on two publicly available hedging datasets. On the fruit-fly dataset, we achieve a precision-recall breakeven point (BEP) of 0.85 using the semi-automatic weighting scheme and a lower BEP of 0.80 with the information gain weighting scheme. These results are competitive with the previously reported best results (BEP of 0.85). On the BMC dataset, using semi-automatic weighting yields a BEP of 0.82, a statistically significant improvement (p <0.01) over the previously reported best result (BEP of 0.76), while information gain weighting yields a BEP of 0.70.

**Conclusion:**

Our results demonstrate that speculative language can be recognized successfully with a linguistically motivated approach and confirms that selection of hedging devices affects the speculative strength of the sentence, which can be captured reasonably by weighting the hedging cues. The improvement obtained on the BMC dataset with a semi-automatic weighting scheme indicates that our linguistically oriented approach is more portable than the machine-learning based approaches. Lower performance obtained with the information gain weighting scheme suggests that this method may benefit from a larger, manually annotated corpus for automatically inducing the weights.

## Background

Scientific method involves making hypotheses, experimenting, and reasoning to reach conclusions, which are often tentative and provisional. Scientific writing, particularly in biomedical research articles, reflects this, as it is rich in speculative statements (hedges). Most text processing systems ignore hedging and focus on factual language (assertions). Although assertions, or even mere co-occurrence of terms, may be sufficient for some information extraction and text mining applications, identifying hedged text is crucial, because hedging acts as a valence shifter, altering, and in some cases even reversing, factual statements.

For instance, the italicized fragment in example (1a) below implies a factual statement while example (1b) is considered speculative:

(1) (a) Each empty cell *indicates that *the corresponding TPase query was not used at the particular stage of PSI-BLAST analysis.

(b) The lack of Cut expression in wild-type ventral cells abutting the D-V boundary indicates *that *D-mib is required for Ser signaling by dorsal cells and acts in a non-autonomous manner to activate N in ventral cells.

These examples not only illustrate the phenomenon of hedging in the biomedical literature, they also highlight difficulties in recognizing hedges. The word *indicate *plays a different role in each example, acting as a hedging cue only in (1b). Hedging in the second sentence seems to be further marked by the subject of *indicate*, *The lack of Cut expression in wild-type ventral cells abutting the D-V boundary*.

The term hedging was introduced into the linguistic literature by Lakoff [1]. He proposed that natural language sentences can be true or false to some extent, contrary to the dominant truth-conditional semantics paradigm of the era. He was mainly concerned with how words and phrases, such as *mainly *and *rather*, make sentences fuzzier or less fuzzy. Palmer [2] identifies *epistemic modality*, which expresses the speaker's degree of commitment to the truth of propositions and is closely linked to hedging. He identifies three types of epistemic modality: *speculatives *express uncertainty, *deductives *indicate an inference from observable evidence, and *assumptives *indicate inference from what is generally known. He focuses mainly on the use of modal verbs in expressing these types. Chafe [3] uses the term *evidentiality *to describe the hedging phenomenon while adopting a narrower definition of hedges, referring only to the linguistic devices that indicate a less than perfect match between a piece of knowledge and a category, such as *about *and *sort of*. Hyland [4] provides a comprehensive account of hedging in scientific articles from a linguistic perspective. He views hedges as polypragmatic devices with an array of purposes (e.g., weakening the force of statement, expressing deference to the reader, signaling uncertainty) and proposes a fuzzy model in which he categorizes scientific hedges by their pragmatic purpose, such as content-oriented hedges, writer-oriented hedges and reader-oriented hedges. He also identifies the principal syntactic realization devices for different types of hedges, including epistemic verbs (verbs indicating the speaker's mode of knowing), adverbs and modal auxiliaries and presents the most frequently used members of these types based on analysis of a corpus of molecular biology articles. He finds that hedges are similarly distributed between abstracts and full-text and that they are most frequent in Results and Discussion sections of scientific articles. DiMarco and Mercer [5] study the intended communicative purpose (dispute, confirmation, use of materials, tools, etc.) of citations in scientific text and show that hedging is used more frequently in citation contexts.

From an NLP perspective, hedging remains an understudied phenomenon. In their investigation of event recognition in news text, Saurí et al. [6] address *event modality *at the lexical and syntactic level by means of SLINKs (subordination links), some of which ("modal", "evidential") indicate hedging. They use corpus-induced lexical knowledge from TimeBank (Pustejovsky et al. [7]) and standard linguistic predicate classifications, and rely on a finite-state syntactic module to identify subordinated events based on the subcategorization properties of the subordinating event.

For the medical field, Friedman et al. [8] discuss uncertainty in radiology reports and their natural language processing system assigns one of five levels of certainty to extracted findings. More recently, there has been increasing interest in the speculative aspect of biomedical text [9-14]. Some of these studies [9-11,14] focus on issues regarding annotating speculation and approach the problem of recognizing speculation as a text classification problem (speculative vs. non-speculative sentences), using machine learning techniques with variants of the well-known "bag-of-words" approach [9,10,13] or simple substring matching [9]. Light et al. [9] obtain slightly better accuracy with substring matching than with an SVM classifier. Medlock and Briscoe [10] extend Light et al.'s work by creating a publicly available hedging dataset and use weakly supervised learning with an SVM classifier to improve to a recall/precision break-even point (BEP) of 0.76, from a BEP of 0.60 obtained using Light et al.'s substring matching method as the baseline. They note that their learning models are unsuccessful in identifying assertive statements of knowledge paucity, generally marked syntactically rather than lexically. Szarvas [13] extends the work of Medlock and Briscoe by performing feature selection, using bi- and trigrams and exploiting external dictionaries. In addition to biomedical research articles, he investigates hedging in radiology reports and finds that they contain mainly unambiguous lexical hedging cues, while scientific articles more commonly contain multi-word hedging cues. Using Medlock and Briscoe's dataset, he obtains a BEP of 0.79 with feature selection and further improves on this result filtering features manually and using external dictionaries (BEP of 0.85). He reports relatively poor results on biomedical articles from a different source (BEP of 0.76) concluding that the portability of hedge classifiers is limited.

A limitation of these machine-learning based approaches is that they do not consider the more complex and subtle ways hedging can occur in biomedical research articles. In example (2), for instance, hedging is achieved in combination with reference to experimental results (*We *... *show that *... *indicating*) and the prepositional phrase *to our knowledge*:

(2) We further show that D-mib is specifically required for Ser endocytosis and signaling during wing development indicating for the first time to our knowledge that endocytosis regulates Ser signaling.

Some annotation studies aim to identify the scope of and the type of information expressed by hedging. For instance, Wilbur et al. [11] recognize that factual information mining is not sufficient and present an annotation scheme in which they propose five qualitative dimensions to characterize scientific sentences, including two that concern hedging: certainty (0–3) and evidence (E0–E3). Szarvas et al. [14] present the BioScope corpus, which consists of medical and biological texts annotated for negation and speculation together with their linguistic scope. Thompson et al. [12] categorize phrases expressing epistemic modality in biomedical text according to the type of information expressed (e.g., level of certainty, point of view, knowledge type) and test the scheme with a small annotation experiment.

### Overview

This paper extends previous work through linguistically motivated techniques. Syntactic structures, in particular, are given special attention. Hyland [4] provides the basic linguistic underpinnings for our study. Our goals are similar to those outlined in Light et al. [9], Medlock and Briscoe [10] and Szarvas [13]; however, we propose that a more linguistically oriented approach can enhance recognition of speculative language, as well as provide the basis for addressing the semantics of speculative language.

We identify lexical hedges from a set of core lexical surface realizations identified in Hyland [4], augmenting this set through analysis of a training set of 521 sentences, 213 of which are speculative. To capture more complex strategic hedges, we identify syntactic patterns that commonly indicate hedging. Furthermore, we identify lexical cues and syntactic patterns that strongly suggest non-speculative contexts ("unhedgers").

We then expand the set of lexical hedging and "unhedging" cues using WordNet [15] and the UMLS SPECIALIST Lexicon [16]. Recognizing that "not all hedges are created equal", we explore quantifying the strength of the hedging cues and patterns with two different methods: semi-automatic (SA) weighting, inspired by the discussion of hedging cues in Hyland [4] and information gain (IG) weighting, based on the information gain measure frequently used in machine learning as a term goodness criterion [17]. We also describe the strengthening or weakening effect of certain syntactic structures on lexical hedging cues. We evaluate our system on two publicly available datasets annotated for hedging.

## Methods

### Data

In our experiments, we use two datasets. One of these datasets is the publicly available hedge classification dataset [10] available at [18], which consists of a manually annotated test set of 1537 sentences (380 speculative) extracted from six full-text articles on *Drosophila melanogaster *(fruit-fly) and a training set of 13,964 sentences (6423 speculative) automatically induced using a probabilistic acquisition model. These sentences are from a pool of 300,000 sentences randomly selected from an archive of 5579 full-text articles. We refer the reader to [10] for details on the annotation guidelines. Good inter-annotator agreement is reported on the test set (original *Cohen's kappa *value (*κ*) of 0.93 and corrected value of 0.98). This dataset is referred to as the fruit-fly dataset in the rest of this paper.

While a probabilistic model for training data acquisition is suitable for the type of weakly supervised learning approach described in [10], it does not yield a representative data sample, because it is noisy and biased towards the hedging cues used as seed terms (*suggest*, *likely*). Their manually annotated test set, however, is valuable for our purposes and is used as one of the test sets here. For a training set, the first author (HK) manually annotated a separate training set of 521 sentences (213 speculative) randomly selected from the pool of sentences in the fruit-fly dataset, using the annotation guidelines provided in [10].

Despite being admittedly small, the training set seems to provide a good sample, as the distribution of surface realization features (epistemic verbs (30%), adverbs (20%), adjectives (16%), modal verbs (23%)) corresponds roughly to that presented in Hyland [4].

In addition to the fruit-fly test set, we tested our system on a recent publicly available hedging dataset [13] available at [19] to determine the portability of our system. This dataset consists of 4 full-text articles published in BMC Bioinformatics and manually annotated according to the guidelines provided in [10]. There are a total of 1087 sentences in this dataset, 190 of which are speculative. We refer to this dataset as the BMC dataset for the rest of this paper.

### Core surface realizations of hedging

Hyland [4] categorizes surface realizations of hedging in scientific articles into two classes: lexical and non-lexical features. Lexical features include modal auxiliaries (*may *and *might *being the strongest indicators), epistemic verbs, adjectives, adverbs and nouns. Some common examples of these feature types are given in Table 1.

**Table 1:** Examples of lexical surface realizations of hedging

**Feature Type**	**Examples**
Modal auxiliaries	*may*, *might*, *could*, *would*, *should*
Epistemic judgment verbs	*suggest*, *indicate*, *speculate*, *believe*, *assume*
Epistemic evidential verbs	*appear*, *seem*
Epistemic deductive verbs	*conclude*, *infer*, *deduce*
Epistemic adjectives	*likely*, *probable*, *possible*
Epistemic adverbs	*probably*, *possibly*, *perhaps*, *generally*
Epistemic nouns	*possibility*, *suggestion*

Non-lexical hedges usually include reference to limiting experimental conditions, reference to a model or theory or admission to a lack of knowledge. Their surface realizations typically go beyond words and even phrases. An example is given in sentence (3), with hedging cues italicized.

(3) Whereas much attention has focused on elucidating basic mechanisms governing axon development, *relatively little is known *about the genetic programs required for the establishment of dendrite arborization patterns that are hallmarks of distinct neuronal types.

While lexical features can be exploited effectively by machine learning approaches, automatic identification of non-lexical hedges automatically requires syntactic and, in some cases, semantic analysis of the text. Our first step was to expand on the core lexical surface realizations identified by Hyland [4].

### Expansion of lexical hedging cues

Epistemic verbs, adjectives, adverbs and nouns provide the bulk of the hedging cues. Although epistemic features are commonly referred to and analyzed in the linguistics literature, the widely used lexicons (e.g., WordNet [15] or, for verb classes, VerbNet [20]) do not systematically indicate epistemological status of the words. We explore inducing a hedging lexicon from the core lexical examples identified in Hyland [4] (a seed list of 63 hedging cues) and expanding it in a semi-automatic manner using two lexicons: WordNet [15] and UMLS SPECIALIST Lexicon [16].

The first step in expanding the hedging lexicon was to extract synonyms for each epistemic term in our seed list using WordNet synsets. We then removed those synonyms that did not occur in our pool of sentences, since they are likely to be quite uncommon words in scientific articles. Expanding epistemic verbs is somewhat more involved than expanding other epistemic types, as they tend to have more synsets, indicating a greater degree of word sense ambiguity (e.g., *assume *has 9 synsets). Based on the observation that an epistemic verb taking a clausal complement marked with *that *is a very strong indication of hedging, we stipulated that only the verb senses which subcategorize for a *that *complement would be considered. Expansion via WordNet resulted in 66 additional lexical features.

Next, we considered the case of nominalizations. Again, based on corpus analysis, we noted that nominalizations of epistemic verbs and adjectives are a common and effective means of hedging in molecular biology articles. The UMLS SPECIALIST Lexicon provides syntactic information, including nominalizations, for biomedical as well as general English terms. We extracted the nominalizations of words in our expanded dictionary of epistemic verbs and adjectives from the UMLS SPECIALIST Lexicon and discarded those that do not occur in our pool of sentences, resulting in an additional 48 terms. Additional five lexical hedging cues (e.g., *tend*, *support*) were identified via data analysis and further expanded using the methodology described above.

An interesting class of cues are terms expressing strong certainty ("unhedgers"). Used within the scope of negation, these terms suggest hedging, while in the absence of negation they strongly suggest a non-speculative context. Examples of these include verbs indicating certainty, such as *know*, *demonstrate*, *prove *and *show*, and adjectives, such as *clear*. These features were also added to the dictionary and used together with negation cues to recognize speculative sentences. The hedging dictionary contains a total of 190 entries.

### Quantifying hedging strength

It is clear that not all hedging devices are equally strong and that the choice of hedging device affects the strength of the speculation. However, determining the strength of a hedging device is not trivial. The fuzzy pragmatic model proposed by Hyland [4] employs general descriptive terms such as "strong" and "weak" when discussing particular cases of hedging and avoids the need for precise quantification. Light et al. [9] report low inter-annotator agreement in distinguishing low speculative sentences from highly speculative ones.

From a computational perspective, it would be beneficial to quantify the strength of hedging cues to determine the confidence of the author in his or her proposition. To this end, we experimented with semi-automatic (SA) weighting and information gain (IG) weighting schemes. Additionally, we accumulate the weights of the hedging cues found in a sentence to assign an overall hedging score to each sentence. This is motivated by the observation in Hyland [4] that writers tend to combine hedges ("harmonic combinations") and the suggestion that scales of certainty and tentativeness could be constructed from these combinations.

#### Semi-automatic (SA) weighting

As a first step in accommodating noticeable differences in strengths of hedging cues, we assigned weights (1 to 5, 1 representing the lowest hedging strength and 5 the highest) to all hedging cues in our dictionary in a semi-automatic manner. The features in the initial seed list were assigned weights manually based on the discussion in Hyland [4]. For instance, he identifies modal auxiliaries, *may *and *might*, as strong prototypical hedging devices, and they were given weights of 5. On the other hand, modal auxiliaries commonly used in non-epistemic contexts (*would*, *could*) were assigned a lower weight of 3. Though not as strong as *may *and *might*, core epistemic verbs and adverbs are generally good hedging cues and therefore were assigned weights of 4. Core epistemic adjectives and nouns often co-occur with other syntactic features to act as strong hedging cues and were assigned weights of 3. Terms added to the dictionary via expansion were assigned a weight one less than their seed terms. For instance, the nominalization *supposition *has weight 2, since it is expanded from the verb *suppose *(weight 3), which is further expanded from its synonym *speculate *(weight 4), a core epistemic verb. The reduction in weights of certain hedging cues is aimed at reflecting their peripheral nature in hedging.

#### Information gain (IG) weighting

We also explored inducing the weights of hedging cues automatically from the training set. For this purpose, we used information gain (IG) measure, often employed in text classification for feature selection. The information gain of a feature *X *with respect to the class label *Y *is defined as "the reduction in uncertainty about the value of *Y *when the value of *X *is known" [17] and is given as

*IG*(*Y*|*X*) = *H*(*Y*) - *H*(*Y*|*X*)

where *H*(*Y*) is the uncertainty about the value of *Y *(the entropy of *Y*) and *H*(*Y*|*X*) is the uncertainty about the value of *Y *when the value of *X *is known (the conditional entropy of *Y *given *X*). *H*(*Y*) is formally defined as

$$H(Y)=-\sum_{i=1}^{k}P(Y=y_{i})log_{2}(P(Y=y_{i}))$$

and *H*(*Y*|*X*) as

$$H(Y|X)=-\sum_{j=1}^{l}P(X=x_{j})H(Y|X=x_{j})$$

where *X *and *Y *are discrete variables. Informally speaking, hedging cues that occur frequently in the speculative sentences but never in non-speculative sentences will have a higher IG weight. To be consistent with the SA weighting scheme, we normalized IG weights of hedging cues to between 1 and 5. The cues that do not appear in the training set are assigned an IG weight of 1.

### The role of syntax

Data analysis reveals that various syntactic devices play a prominent role in hedging, both as hedging cues on their own and for strengthening or weakening effects. For instance, while some epistemic verbs do not act as hedging cues (or may be weak hedging cues) when used alone, together with a *that *complement or an infinitival clause, they become good indicators of hedging. A good example is *appear*, which often occurs in molecular biology articles with its non-speculative "come into sight" meaning (4a) but becomes a good hedging cue when it takes an infinitival complement (4b):

(4) (a) The linearity of the ommatidial arrangement was disrupted and numerous gaps *appeared *between ommatidia arrow.

(b) In these data a substantial fraction of both silent and replacement DNA mutations *appear *to affect fitness.

On the other hand, as discussed above, words expressing strong certainty ("unhedgers") are good indicators of hedging only when negated, and are strongly non-speculative otherwise.

We examined the training set and identified the most salient syntactic patterns that play a role in hedging and their contribution to hedging strength. These patterns and their effect on the overall hedging score are given in Table 2. Syntactic patterns may contribute to hedging strength in two ways: as a) conditional triggers and b) absolute triggers. As a conditional trigger, a syntactic pattern, or lack thereof, strengthens or weakens the lexical hedging cue that is involved in it; a strengthening syntactic pattern will increase the weight contributed by the cue, while a weakening pattern will decrease it. For instance, in example (4a) above, the absence of the infinitival complement will reduce the score contribution of *appear *by 1, resulting in a score of 3 instead of 4. On the other hand, the infinitival complement in example (4b) will increase the score contribution of *appear *by 1. In case of absolute triggers, the existence of a particular syntactic pattern is sufficient to render the sentence speculative (independent of any lexical hedging cues). One such case we identified is that of *whether *(*if*): it acts as a hedging cue when it introduces a clausal complement regardless of existence of any other hedging cue from the hedging dictionary. *whether *(*if*) was assigned a weight of 3 in SA weighting scheme.

**Table 2:** Syntactic patterns and their effect on hedging strength

**Syntactic Pattern**	**Effect on strength**
<EPISTEMIC VERB> *to*(*inf*) VB	+1
<EPISTEMIC VERB> *that*(*comp*) VB	+2
Otherwise	-1
<EPISTEMIC NOUN> followed by *that*(*comp*)	+2
Otherwise	-1
*not *<UNHEDGING VERB>	+1
*no*|*not *<UNHEDGING NOUN>	+2
*no*|*not *immediately followed by <UNHEDGING ADVERB>	+1
*no*|*not *immediately followed by <UNHEDGING ADJECTIVE>	+1
*whether*|*if *in a clausal complement context	3(SA) 1.58(IG)

To obtain the syntactic structures of sentences, we use the statistical Stanford Lexicalized Parser [21], which provides a full parse tree, in addition to part-of-speech tagging based on the Penn Treebank tagset. A particularly useful feature of the Stanford Lexicalized Parser is typed dependency structures extracted from phrase structure parses [22]. We use these typed dependency parses to identify clausal complements, infinitival clauses, and negation. For instance, the dependency relations in (5) below indicate a clausal complement marked with *that *and identify the second syntactic pattern in Table 2.

(5) *ccomp*(<*EPISTEMIC VERB*>,<*VB*>)

*complm*(<*VB*>,*that*)

In these dependency relations, *ccomp *stands for clausal complement with internal subject and *complm *stands for complementizer, *VB *indicates any verb.

### Baseline methods

We compared our system with two baseline methods. The first baseline method (baseline1) uses the substring matching method reported in Light et al. [9], which labels sentences containing one or more of the following as speculative: *suggest*, *potential*, *likely*, *may*, *at least*, *in part*, *possibl*, *further investigation*, *unlikely*, *putative*, *insights*, *point toward*, *promise and propose*. Similarly, the second baseline method (baseline2) uses substring matching, with the top 15 ranked term features determined using *P*(*spec*|*x*_*j*_) in training and classification models (at smoothing parameter *α *= 5) reported in Medlock and Briscoe [10]: *suggest*, *likely*, *may*, *might*, *seems*, *Taken*, *suggests*, *probably*, *Together*, *suggesting*, *possibly*, *suggested*, *findings*, *observations*, *Given*.

## Results

We evaluate our method on the fruit-fly and BMC datasets, using basic information retrieval evaluation metrics: precision, recall, accuracy and F_1 _score. In addition, we measure the recall/precision break-even point (BEP), which indicates the point at which precision and recall are equal, to provide a comparison to results previously reported. Our system computes an overall hedging score for each sentence by summing up the weights of hedging indicators involved. We evaluate our system by using this overall score as threshold to control the precision/recall balance. To measure the statistical significance of performance differences between our system and the baseline methods, we use the binomial sign test.

### Evaluation with the fruit-fly dataset

The baseline methods yield the evaluation results given in Table 3 on the fruit-fly dataset. The evaluation results using SA weighting on this dataset are given in Table 4. Note that the highest overall hedging score we obtained with this weighting scheme is 16; however, we do not show the results for every possible threshold here for brevity.

**Table 3:** Evaluation results of the baseline methods using the fruit-fly dataset

**Method**	**Precision**	**Recall**	**Accuracy**	**F_1 _score**
baseline1 (14 strings)	0.79	0.40	0.82	0.53
baseline2 (15 strings)	0.95	0.43	0.85	0.60

**Table 4:** Evaluation results from our system using SA weighting on the fruit-fly dataset

**Threshold**	**Precision**	**Recall**	**Accuracy**	**F_1 _score**
1	0.68	0.95	0.88	0.79
2	0.74	0.94	0.90	0.83
3	0.85	0.86	0.93	0.85
4	0.91	0.71	0.91	0.80
5	0.92	0.63	0.89	0.75
6	0.97	0.40	0.85	0.57
7	1	0.19	0.79	0.33

Tables 3 and 4 show that the results obtained by our method with SA weighting improve on both baseline methods in terms of accuracy and F_1 _score. Increasing the threshold (thereby requiring more or stronger hedging devices to qualify a sentence as speculative) improves the precision while lowering the recall. The best accuracy and F_1 _score are achieved at threshold t = 3. At this threshold, the differences between the results obtained with our method and the baseline methods are both statistically significant at 0.01 level (*p *< 0.01).

Performing the same experiment with IG weighting, we obtain the results given in Table 5. The highest overall hedging score in this case is 12.95. The best accuracy and F_1 _score are obtained with threshold t = 1.5. While the improvement over the baseline methods is less pronounced with this weighting scheme, the differences between the results obtained with this method at t = 1.5 and the baseline methods are similarly both statistically significant (*p *< 0.01).

**Table 5:** Evaluation results from our system using IG weighting on the fruit-fly dataset

**Threshold**	**Precision**	**Recall**	**Accuracy**	**F_1 _score**
1	0.66	0.89	0.86	0.76
1.5	0.81	0.79	0.90	0.80
2	0.83	0.69	0.89	0.75
3	0.88	0.53	0.87	0.66
5	0.98	0.25	0.81	0.40
6	1	0.13	0.79	0.24

With SA weighting, the best threshold (t = 3) provides roughly equal precision and recall, indicating a recall/precision BEP of approximately 0.85, a significant improvement over 0.76 achieved with a weakly supervised classifier [10] and a result roughly equivalent to that achieved with a weakly supervised classifier with feature selection and external dictionaries [13]. Despite being lower than that obtained with SA weighting, recall/precision BEP obtained with the IG weighting scheme (0.80) is still an improvement over those obtained with baseline methods and with the weakly supervised classifier [10].

### Evaluation with the BMC dataset

In addition to the fruit-fly dataset, we evaluated our system on the smaller BMC dataset. The evaluation results obtained using the baseline methods on this dataset are given in Table 6. The SA weighting scheme gives the evaluation results in Table 7. The highest overall hedging score obtained with SA weighting is 9. Tables 6 and 7 reveal that, on a different dataset, our system is able to give good results, with the best result again coming at threshold t = 3. Using the IG weighting scheme on this dataset, we obtain the results given in Table 8. The best result in this case is obtained with threshold t = 1.75. The highest hedging score obtained in this case is 10.90. For the best threshold (t = 3), the SA weighting scheme provides close precision and recall figures, indicating a recall/precision BEP of approximately 0.82. On the other hand, the IG weighting scheme gives relatively poor results on this dataset, providing an approximate BEP of 0.70. All recall/precision BEP scores are given in Table 9.

**Table 6:** Evaluation results of the baseline methods using the BMC dataset

**Method**	**Precision**	**Recall**	**Accuracy**	**F_1 _score**
baseline1 (14 strings)	0.65	0.52	0.87	0.58
baseline2 (15 strings)	0.83	0.47	0.89	0.60

**Table 7:** Evaluation results from our system using SA weighting on the BMC dataset

**Threshold**	**Precision**	**Recall**	**Accuracy**	**F_1 _score**
1	0.58	0.96	0.87	0.73
2	0.66	0.94	0.90	0.77
3	0.80	0.85	0.94	0.82
4	0.83	0.65	0.92	0.73
5	0.95	0.56	0.92	0.70
6	0.97	0.35	0.89	0.52
7	0.98	0.21	0.86	0.35

**Table 8:** Evaluation results from our system using IG weighting on the BMC dataset

**Threshold**	**Precision**	**Recall**	**Accuracy**	**F_1 _score**
1	0.48	0.82	0.81	0.60
1.5	0.66	0.74	0.89	0.70
1.75	0.75	0.67	0.90	0.71
2	0.75	0.65	0.90	0.70
2.5	0.88	0.54	0.91	0.67
5	0.97	0.35	0.89	0.52

**Table 9:** Recall/precision break-even point (BEP) results

**Method**	**Recall/Precision BEP**
baseline1	0.60
baseline2	0.76
Our system on the fruit-fly dataset with SA weighting	0.85
Our system on the fruit-fly dataset with IG weighting	0.80
Our system on the BMC dataset with SA weighting	0.82
Our system on the BMC dataset with IG weighting	0.70

## Discussion

Our results confirm that writers of scientific articles employ basic, predictable hedging strategies to soften their claims or to indicate uncertainty. Moreover, they demonstrate that these strategies can be captured using a combination of lexical and syntactic means. Furthermore, the results indicate that hedging cues can be gainfully weighted to provide a rough measure of tentativeness or speculation. For instance, a sentence with one of the highest overall hedging scores (ranked highest with SA weighting and fourth highest with IG weighting) is given in example (6):

(6) In one study, Liquid facets was *proposed *to target Dl to an endocytic recycling compartment *suggesting that *recycling of Dl *may *be required for signaling.

On the other hand, hedging is not strong in example (7), and both weighting schemes demonstrate this point (the sentence ranked second lowest with SA weighting and fourth lowest with IG weighting).

(7) There is *no apparent *need for cytochrome c release in C. elegans since CED-4 does not require it to activate CED-3.

Overall, the SA weighting scheme gives better results. There may be two factors: First, even though information gain is a good indicator of the discriminatory power of a hedging cue for a given dataset, it may not act as a reliable measure of its overall hedging strength. For instance, *may *is assigned the highest weight by both SA and IG weighting, confirming the intuition that it is the prototypical hedging device in research articles; on the other hand, a strong epistemic verb like *speculate *is given a low IG weight, since it occurs infrequently in the training data set, albeit always in hedged sentences. This brings us to the second factor, that is, the size of the training dataset. It is irrelevant to SA weighting. On the other hand, a small training set makes it difficult to automatically induce weights based on the frequency of occurrence of hedging cues. In fact, in our training set, some hedging cues do not appear at all. It is reasonable to argue that a larger training set will yield a more accurate weighting scheme based on IG measure. However, the superior results obtained with SA weighting confirm our intuition that a weighting scheme relying on the particular semantic properties of the indicators is likely to capture the hedging strengths more accurately.

Comparison of results from the fruit-fly and BMC datasets shows that SA weighting provides relatively stable results across datasets. (BEP of 0.85 vs. BEP of 0.82). This is in contrast with the finding in [13] that hedging cues are task-specific and not portable, based on the results he obtains (BEP of 0.85 vs. BEP of 0.76) and points to the possibility that our system with SA weighting scheme is more generalizable than one based on machine learning techniques. It is also interesting to note that our system performs poorly on the BMC dataset with the IG weighting scheme (BEP of 0.70), suggesting that the portability of our system does not extend to this type of weighting.

### Error analysis

Below, we discuss some of the common error types we encountered. Our discussion is based on evaluation at a hedging score threshold of 0, where existence of a single hedging cue is sufficient to label a sentence speculative.

Most of the false negatives produced by the system are due to syntactic patterns not addressed by our method. For instance, negation of "unhedgers" was used as a syntactic pattern; while this pattern correctly identified *know *as an "unhedger" in the following sentence, it did not recognize *little *as a negative quantifier, consequently labeling the sentence as non-speculative.

(8) *Little was known *however about the specific role of the roX RNAs during the formation of the DCC.

In fact, Hyland [4] notes that "negation in scientific research articles shows a preference for negative quantifiers (*few*, *little*) and lexical negation (*rarely*, *overlook*)." However, we have not encountered this pattern while analyzing the training set and have not addressed it. Nevertheless, our approach lends itself to incremental development and adding such a pattern to our rulebase is relatively simple.

Another type of false negative is caused by certain derivational forms of epistemic words. In the following example, the adjective *suggestive *is not recognized as a hedging cue, even though its base form *suggest *is an epistemic verb.

(9) Phenotypic differences are *suggestive *of distinct functions for some of these genes in regulating dendrite arborization.

More sophisticated lexicon expansion rules can be employed to handle these cases, such as WordNet's "derivationally related form" feature.

Even though the Stanford Lexicalized Parser is not customized for the biomedical domain, we found that, in general, it accurately identifies the limited number of syntactic patterns we are interested in. One of the rare errors caused by incorrect dependency relations is given in example (10):

(10) *Whether *the codon aligned to the inframe stop codon *is *a nonsense codon or not *was *neglected at this stage.

In this sentence, *aligned *rather than *is *is identified as the predicate of the clause introduced by *Whether*. Most of the false positives are due to word sense ambiguity of hedging cues. For instance, the modal auxiliary *could *is frequently used as a past tense form of *can *in scientific articles to express the role of enabling conditions and external constraints on the occurrence of the proposition rather than uncertainty or tentativeness regarding the proposition. Currently, our system is unable to recognize cases such as (11):

(11) Also we *could *not find any RAG-like sequences in the recently sequenced sea urchin lancelet hydra and sea anemone genomes, which encode RAG-like sequences.

Context around a hedging cue plays a role in these cases. First person plural pronoun (*we*) and/or reference to objective enabling conditions seem to be a common characteristic among false positive cases of *could*.

For cases such as *appear*, where the absence of strengthening complement clauses (*to*, *that*) lowers the hedging score, the threshold may be too high to render the sentence non-speculative. Rather than treating all epistemic verbs equally, a more appropriate approach would be to consider verb senses separately (e.g., *appear *should be effectively unhedged without a strengthening cue, while *suggest *should only be weakened).

Another type of false positives concern "weak" hedging cues, such as epistemic deductive verbs (*conclude*, *estimate*) as well as some adverbs (*essentially*, *usually*) and nominalizations (*implication*, *assumption*).

We have also seen a few controversial instances, which seem speculative on the surface, but were labeled non-speculative. An example from the fruit-fly dataset is given in example (12):

(12) Caspases can also be activated with the aid of Apaf-1, which in turn *appears *to *be *regulated by cytochrome c and dATP.

## Conclusion

This paper presents experiments we conducted in recognizing speculative sentences. We draw on previous linguistic work, extend it via semi-automatic lexical acquisition methods and weighting of hedging cues. Using two datasets specifically annotated for speculation, we demonstrate that our linguistically oriented approach improves on or gives results competitive with the previously reported results. Semi-automatic weighting scheme captures the speculative strength of hedging cues more accurately.

Our next goal is to extend our work using a larger, more comprehensive corpus. This will allow us to identify other commonly used hedging strategies and refine and expand the hedging dictionary. A larger training corpus could also allow us to refine our weighting schemes.

While recognizing that a sentence is speculative is useful in and of itself, it seems more interesting and clearly much more challenging to identify speculative sentence fragments and the scope of hedging devices. We plan to move in this direction with the goal of characterizing the semantics of speculative language.

## Competing interests

The authors declare that they have no competing interests.

## Authors' contributions

HK conceived of the study, performed the analyses and programming, and drafted the manuscript. SB participated in the design of the study and helped draft the manuscript. Both authors read and approved the final manuscript.
